# Apoptosis Induced by Cytoskeletal Disruption Requires Distinct Domains of MEKK1

**DOI:** 10.1371/journal.pone.0017310

**Published:** 2011-02-25

**Authors:** Erin Tricker, Afsane Arvand, Raymond Kwan, Gordon Y. Chen, Ewen Gallagher, Genhong Cheng

**Affiliations:** 1 Department of Microbiology, Immunology & Molecular Genetics, University of California Los Angeles, Los Angeles, California, United States of America; 2 Department of Biology, Mount Saint Mary's College, Los Angeles, California, United States of America; 3 Department of Immunology, Imperial College London, London, United Kingdom; Wayne State University School of Medicine, United States of America

## Abstract

MEKK1 is a mitogen-activated protein kinase kinase kinase (MAP3K) that activates the MAPK JNK and is required for microtubule inhibitor-induced apoptosis in B cells. Here, we find that apoptosis induced by actin disruption via cytochalasin D and by the protein phosphatase 1/2A inhibitor okadaic acid also requires MEKK1 activation. To elucidate the functional requirements for activation of the MEKK1-dependent apoptotic pathway, we created mutations within MEKK1. MEKK1-deficient cells were complemented with MEKK1 containing mutations in either the ubiquitin interacting motif (UIM), plant homeodomain (PHD), caspase cleavage site or the kinase domain at near endogenous levels of expression and tested for their sensitivity to each drug. We found that both the kinase activity and the PHD domain of MEKK1 are required for JNK activation and efficient induction of apoptosis by drugs causing cytoskeletal disruption. Furthermore, we discovered that modification of MEKK1 and its localization depends on the integrity of the PHD.

## Introduction

MEKK1 is a serine-threonine kinase that activates the c-Jun amino terminal (N-terminal) kinases (JNK) MAPK module, altering gene transcription [1]. We have previously shown that MEKK1 is essential for microtubule inhibitor (MTI)-induced JNK activation and apoptosis through genetic deletion of *MEKK1* in chicken bursal B cells (DT40 cell line) [2]. Given that MTI drugs are important chemotherapeutic agents involved in treatment of leukemia, lymphomas, non-small cell lung cancer, breast cancer, testicular cancer and head and neck cancer, understanding MEKK1 activation within the context of apoptosis will improve chemotherapy regimens and outcomes [3], [4]. The importance of the MEKK1-dependent pathway of apoptosis has recently been highlighted by Kan et al., who identified MEKK1 as one of the top 50 genes containing somatic missense and nonsense mutations in a panel of breast, lung, ovarian and prostate tumors, indicating that impairment of the MEKK1-dependent apoptotic pathway may enhance tumorigenesis [5].

The extent of MEKK1 involvement in apoptosis by other mechanisms of cytoskeletal disruption is currently unknown. Cytochalasin toxins and the protein phosphatase inhibitor okadaic acid are two examples of drugs that disrupt the cytoskeleton and cause JNK activation [6], [7]. Cytochalasins disrupt actin filament integrity by capping the barbed end of actin, thus preventing the addition of G-actin monomers [8]. Okadaic acid inhibits protein phosphatase 1 and 2A and results in the rapid phosphorylation and disruption of intermediate filaments [9]. Whether the MEKK1-dependent apoptotic pathway is involved in the response to cytochalasins or protein phosphatase inhibition has not been investigated.

MEKK1 is a 196 kDa protein comprised of a large N-terminal regulatory region and a C-terminal kinase domain. The N-terminal region contains features such as a plant homeodomain (PHD), two overlapping ubiquitin-interacting motifs (UIM), and a caspase cleavage site [10], [11]. Several studies have investigated the potential functions of different regions of MEKK1. The PHD is a multifunctional domain that is involved in protein interactions and is an E3 ligase. An intact PHD is required for interaction with RhoA, a small GTPase [12]. The PHD is also an E3 ligase for ERK (Extracellular regulated Kinases) and c-Jun under hyperosmotic conditions, and is required for MEKK1 autoubiquitination in overexpression conditions and after CD40 ligand stimulation [11], [13], [14], [15]. Another MEKK1 region with a potential role in protein modification by ubiquitin is the UIM. The UIM may aid in the polyubiquitination of MEKK1, as this domain has been required for ubiquitination of other UIM-containing proteins [16].

MEKK1 kinase activity has been correlated to apoptosis. Constitutive kinase activity has been observed when full length MEKK1 is cleaved at its caspase recognition site, and mutation of this site inhibits apoptosis normally seen with overexpression in 293T cells while leaving JNK activation unaffected [17]. In addition, overexpression of MEKK1 with a mutated kinase domain inhibits apoptosis by UV light [18]. However, UV-induced apoptosis is unaffected by the genetic loss of *MEKK1*, highlighting the difference between the dominant negative effects caused by overexpression studies and what occurs under normal physiological conditions [19]. By reconstituting MEKK1-deficient (*MEKK1^−/−^*) cells at more physiologically relevant levels, we can better differentiate any dominant negative effects of these mutations from the role that each may play within the MEKK1-dependent pathway of apoptosis.

Here we show that okadaic acid-induced apoptosis and cytochalasin-induced apoptosis are both impaired by genetic deletion of *MEKK1*. This indicates that MEKK1-dependent apoptosis is not specifically confined to microtubule inhibition, but may play a broader role in apoptosis activated by the two other cytoskeletal structures of the cell: actin fibers and intermediate filaments. Furthermore, we reveal that MEKK1-deficient murine B cells are also resistant to apoptosis induced by cytoskeletal disruption, indicating that this phenotype is not specific to DT40 cells. To determine the role of the various domains within MEKK1, we created mutations within the PHD, UIM, caspase cleavage site and the kinase domain, and MEKK1-deficient cells were reconstituted with each mutant at near endogenous expression levels. Using this system we found that the PHD and the kinase domain are required for MEKK1-dependent JNK activation and apoptosis. Further investigation indicates that mutation of the PHD results in altered subcellular localization and reduced levels of basal ubiquitination found on MEKK1. We also found that while the PHD mutant can be phosphorylated and activate downstream signals when overexpressed at high levels, low level reconstitution of knockout cells with the PHD mutant results in lack of MEKK1 phosphorylation after vinblastine treatment. This suggests that the defect in the PHD mutant resides upstream of MEKK1. Therefore, the mislocalization of the PHD mutant may interfere with its ability to interact with upstream proteins.

## Results

### MEKK1 is required for JNK activation and the apoptotic response to cytoskeletal disruption

**Figure 1:**
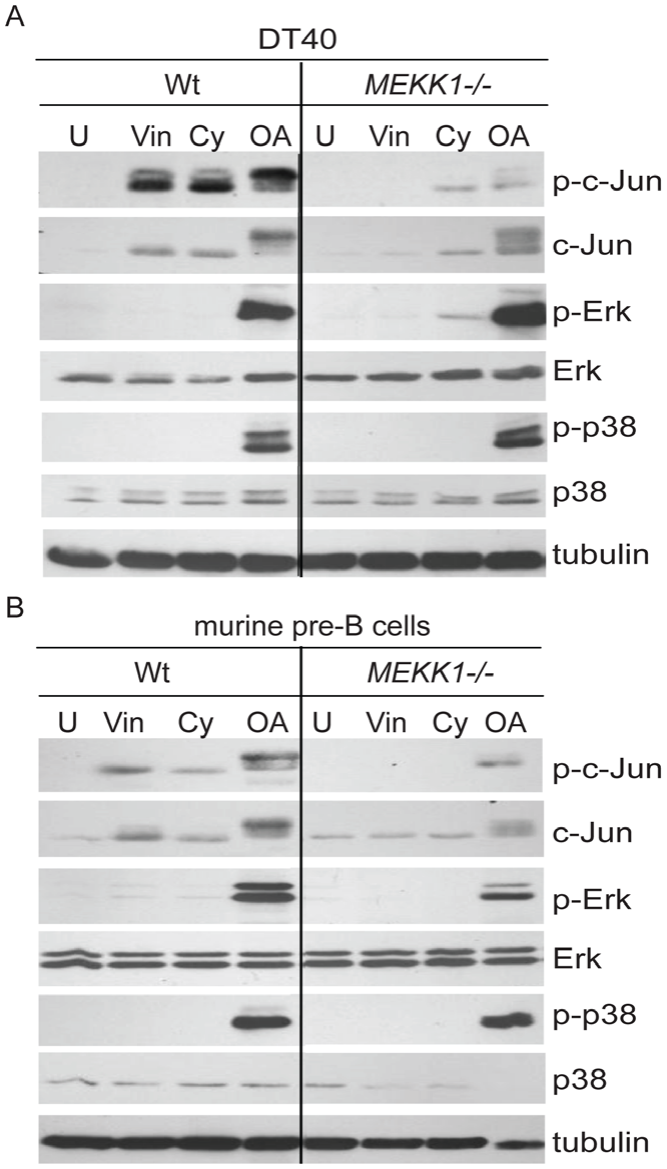
Characterization of MAPK activation in wild type and MEKK1-deficient cell lines. *A*. Phosphorylation of c-Jun phosphorylation is defective in the *MEKK1* ^−/−^ chicken DT40 cells in response to cytoskeletal disruption. Wild type (Wt) and *MEKK1* ^−/−^ DT40 cells were left untreated (U) or stimulated with 1 µM vinblastine (Vin), 2 µg/ml cytochalasin D (Cy), and 90 nM okadaic acid (OA) for four hours, lysed in modified RIPA buffer and run on an SDS-PAGE gel to compare wild type and knockout cell lines. Western blot membranes were probed for phosphorylated and total c-Jun, ERK and p38. *B*. Phosphorylation of c-Jun is defective in *MEKK1* ^−/−^ murine pre-B cells in response to cytoskeletal disruption. Wild type (Wt) and *MEKK1* ^−/−^ pre-B murine cells were stimulated with 1 µM vinblastine, 2 µg/ml cytochalasin D, and 90 nM okadaic acid for four hours, lysed in modified RIPA buffer and run on an SDS-PAGE gel. Membranes were probed for phosphorylated and total c-Jun, ERK and p38. β-tubulin is used as loading control.

Drugs such as vinblastine, cytochalasin D, and okadaic acid all induce cytoskeletal disruption and strong MAPK activation. To determine whether this activation requires MEKK1, DT40 chicken bursal B cell line and its previously generated MEKK1-deficient (*MEKK1^−/−^*) counterpart were treated with vinblastine, cytochalasin D and okadaic acid (Figure 1A). Upon treatment with vinblastine, cytochalasin D and okadaic acid, MEKK1-deficient cells had undetectable or decreased levels of c-Jun phosphorylation, whereas ERK and p38 phosphorylation were unaffected by MEKK1 deficiency. We also noted that total c-Jun levels were elevated after treatment with these drugs, in a manner partially dependent on MEKK1. To ensure that the requirement for MEKK1 in vinblastine-induced apoptosis is not a property inherent to the chicken DT40 cell line, murine wild type or MEKK1-deficient bone marrow cells were transformed to create stable pre-B cell lines. These newly created cell lines were then analyzed for MAPK activation in response to vinblastine, cytochalasin D or okadaic acid. Similar to the DT40 MEKK1-deficient cell line, decreased levels of phosphorylated c-Jun were observed in the murine MEKK1-deficient line as compared with the wild type cells (Figure 1B).

**Figure 2:**
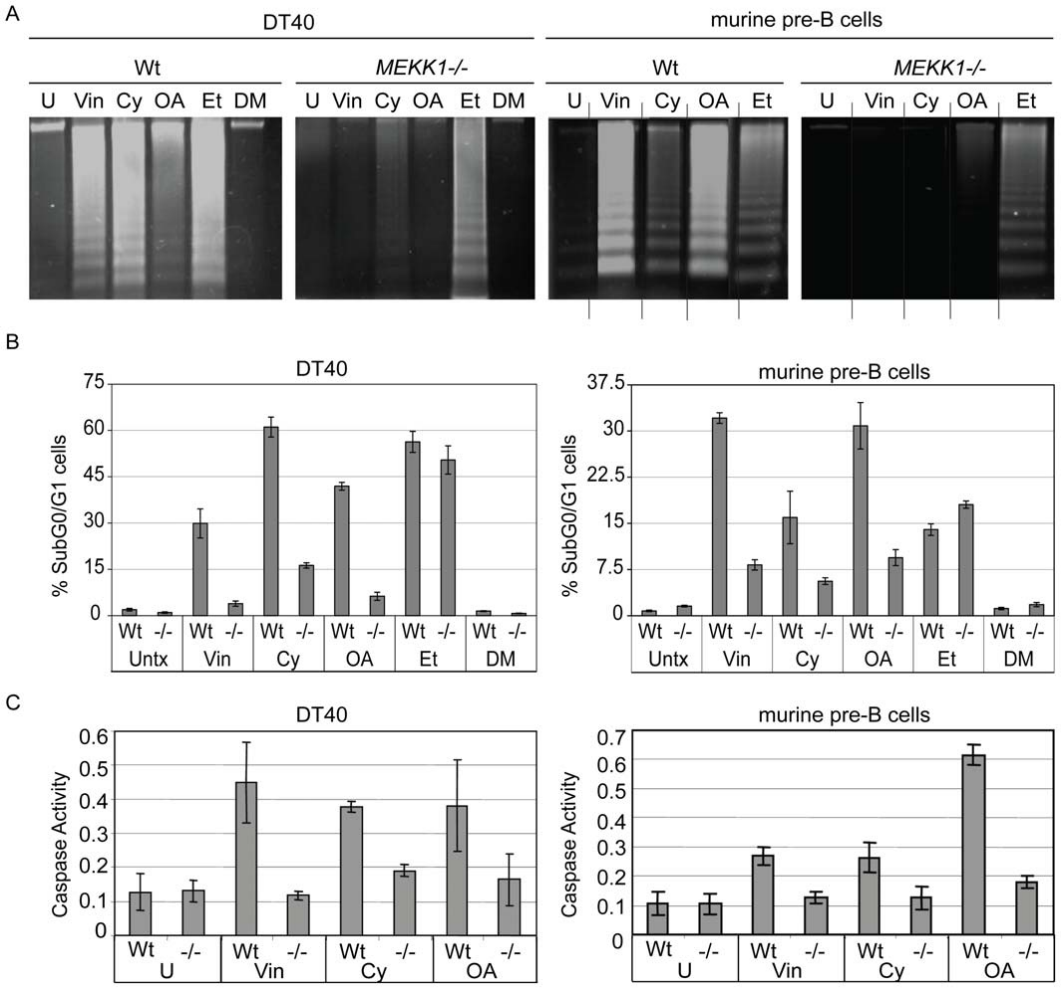
Detection of apoptosis after cytoskeletal disruption in wild type and MEKK1-deficient cell lines. *A*. DNA laddering is significantly decreased in the *MEKK1^−/−^* DT40 and murine pre-B cell lines upon treatment with vinblastine, cytochalasin D, and okadaic acid, whereas the response to etoposide is unchanged. Wild type (Wt) or *MEKK1^−/−^* DT40 cell lines were treated with 1 µM vinblastine (Vin), 2 µg/ml cytochalasin D (Cy), 90 nM okadaic acid (OA), or DMSO (DM) for six hours or 25 µM etoposide (Et) for four hours. After treatment, each line was assessed for the presence of DNA laddering. Wild type (Wt) or *MEKK1−/−* murine cell lines were treated with the same concentration of these drugs for nine hours and DNA laddering was assessed. *B*. Apoptosis was quantified by propidium iodide exclusion assay. Wild type (Wt) or *MEKK1^−/−^* DT40 were treated with 1 µM vinblastine (Vin), 2 µg/ml cytochalasin D (Cy) for DT40 cells, 90 nM okadaic acid (OA), DMSO (DM) or 15 µM etoposide (Et) for 12 hours. Wild type (Wt) or *MEKK1^−/−^* murine pre-B cells were treated with 1 µM vinblastine (Vin), 4 µg/ml cytochalasin D (Cy) for DT40 cells, 90 nM okadaic acid (OA), DMSO (DM) or 15 µM etoposide (Et) for 14 hours. After treatment, the percentage of apoptotic cells was assessed by measuring fluorescence of the sub-diploid population using flow cytometry. *C*. Caspase 3 activation is abrogated in the *MEKK1* ^−/−^ cell lines after cytoskeletal disruption. DT40 and *MEKK1^−/−^* cells (left panel) were treated with 1 µM vinblastine and 2 µg/ml cytochalasin for four hours and 90 nM okadaic acid for six hours. Murine wild type and *MEKK1^−/−^* pre-B cells (right panel) were treated with 1 µM vinblastine and 2 µg/ml cytochalasin and 90 nM okadaic acid for six hours. Cell lysates were used to determine the catalytic activity of caspase 3.

Next, we investigated whether apoptosis induced by these drugs requires MEKK1. Strong DNA laddering was observed in the wild type DT40 and murine B cell lines in response to vinblastine, cytochalasin D and okadaic acid (Figure 2A). However, reduced levels of laddering were seen in both chicken and murine knockout cell lines. This effect did not appear to be a general defect in apoptosis, as treatment with the DNA damaging agent etoposide induced apoptosis in all cell lines (lane Et, Figure 2A). Apoptosis in response to each drug was then quantified by PI exclusion assay for each cell line (Figure 2B). In addition, we assessed caspase 3 activity to show that cells were undergoing apoptosis and not just DNA damage, and found that there is an increased level of active caspase 3 after each drug treatment in the wild type cells when compared to the knockout cells, which is consistent with the DNA laddering and PI exclusion data (Figure 2C). Taken together, these data indicate that MEKK1 is involved in both JNK activation and the apoptotic response to a number of cytoskeletal disrupting agents.

### Overexpression of the PHD mutant MEKK1 affects basal modification and localization, but not induction of downstream signaling

**Figure 3:**
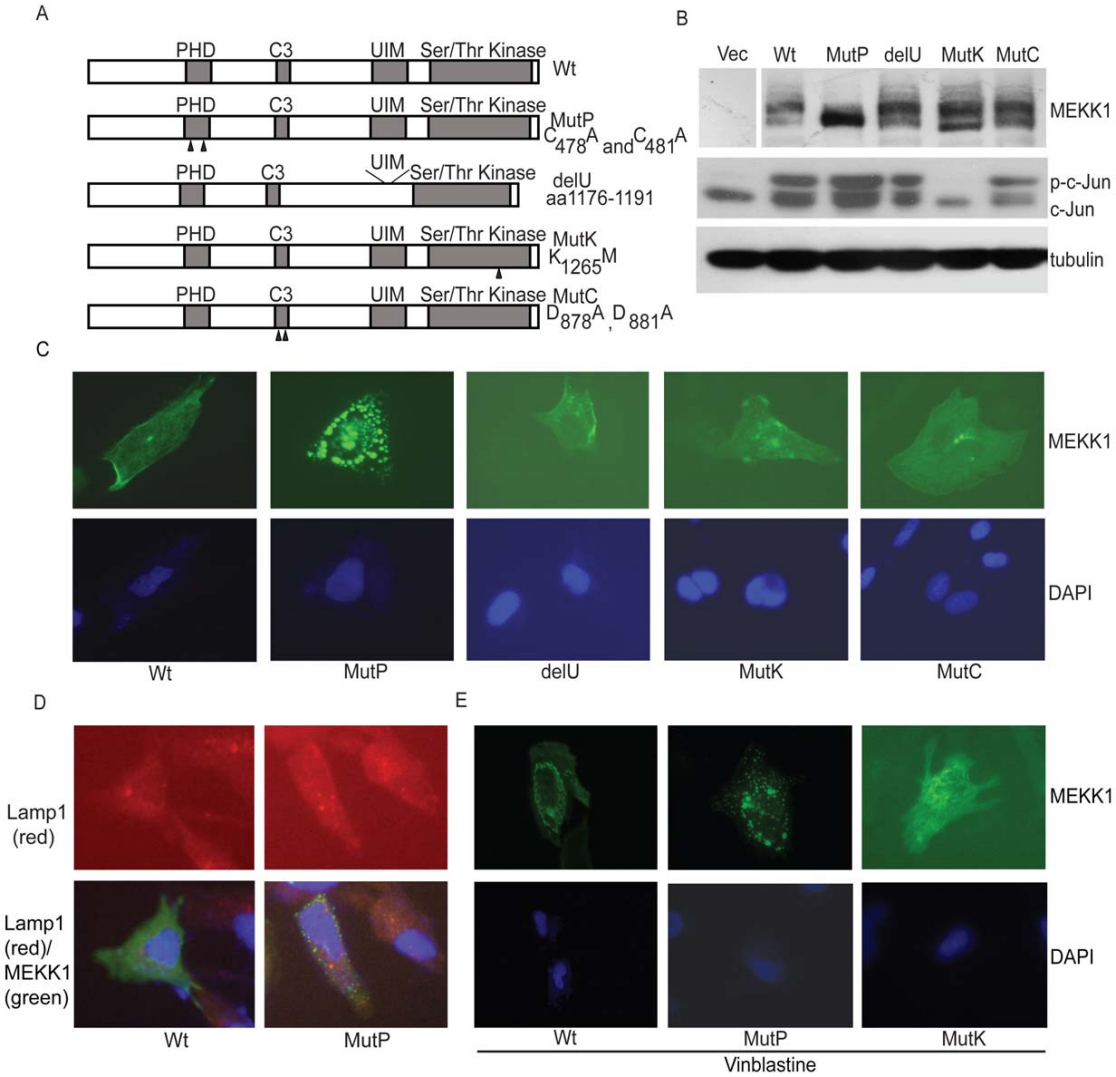
Creation and characterization of MEKK1 mutations. *A*. Schematic diagram of MEKK1, and MEKK1 mutants illustrating the plant homeodomain (MutP), the ubiquitin-interacting motif (delU), the kinase domain (MutK) and the putative caspase cleavage site (MutC). Arrows indicate the mutations created within each domain. *B*. Proper protein expression and determination of downstream signaling via c-Jun phosphorylation after overexpression of each mutation. Each mutant was transiently transfected into 293T cells in the highly expressing pApuro construct. Cell lysates were run on SDS-PAGE, western blots were performed and MEKK1 was detected with αMEKK1 C-22. Blots were next probed with total c-Jun, which recognizes both the dephosphorylated and phosphorylated forms of c-Jun. *C*. Cellular localization of each mutant. HeLa cells were transfected with pApuro MEKK1, MutP, delU, MutK or MutC and stained with DAPI (nucleus) and αMEKK1 C-22 antibody (MEKK1) after 24 hours of transfection. *D*. The PHD mutant does not colocalize with lysosomes to a greater extent than wild type MEKK1. HeLa cells were transfected with flag-MEKK1 or flag-MutP and stained with DAPI, M2 αflag (MEKK1) and LAMP1 (lysosomes) after 24 hours. MEKK1 was detected with mouse αFITC and LAMP1 with rabbit αTRITC. *E*. The PHD mutant localization is not altered by vinblastine treatment. HeLa cells were transfected with pApuro MEKK1, MutP and MutK. After 24 hours cells were treated with vinblastine for one hour. MEKK1 was visualized with αMEKK1 C-22 antibody and nuclei visualized with DAPI.

To identify specific domains of MEKK1 required for apoptosis in response to cytoskeletal disruption, mutations were created within different domains of MEKK1 (Figure 3A). Two mutations were introduced into the PHD domain of rat MEKK1 (^Cys^478^Ala^ and ^Cys^481^Ala^; MutP); these mutations have been shown to disrupt the zinc finger structure and destroy its E3 ligase function [11]. The putative UIM of MEKK1 (amino acid 1176 to 1191) was deleted to create delU. The critical lysine residue within the kinase active site of MEKK1 was mutated to methionine (1265^K^ to 1265^M^; MutK) to disrupt activation of the kinase domain. To determine if caspase cleavage of MEKK1 is required for apoptosis in our system, we mutated the aspartic acid residues in the caspase recognition site of rat MEKK1 to glutamate (^878^DTLD^881^ to ETLE) to create MutC; the orthologous caspase mutation of murine MEKK1 has been reported to render murine MEKK1 resistant to cleavage at this site [17], [20].

Each MEKK1 mutant was overexpressed in 293T cells in the highly expressing pApuro vector and analyzed via western blot to check for protein expression. As seen in Figure 3B, the mutant constructs expressed at the proper MEKK1 protein size of 196 kD. We also noted that normally MEKK1 appears to be highly modified as apparent by the presence of multiple MEKK1 bands (Figure 3B), but among our mutations, the PHD mutant was noticeably lacking this modification.

Because overexpression of MEKK1 causes JNK activation and downstream c-Jun phosphorylation, we next investigated the effects of each mutant in downstream signaling through JNK in our 293T overexpression system. Each mutant could still initiate downstream signaling, as assessed by phosphorylated c-Jun, with the anticipated exception of the kinase mutation (Figure 3B).

To determine the cellular localization of each mutant, we expressed each in HeLa cells and visualized localization with indirect immunofluorescence (Figure 3C). The localization of MEKK1 has been well characterized by immunofluorescence. As previously published and in our system, MEKK1 was expressed diffusely through the cytosol and in small punctuate structures [21]. The UIM, caspase 3 and kinase dead mutants were expressed in a manner similar to wild type MEKK1, but the PHD mutant was located expressly in large, distinct punctuate structures. To ensure that the change in the PHD mutant localization was not due to enhanced lysosomal degradation, indirect immunofluorescence was performed with both MEKK1 and LAMP1, a marker for lysosomes (Figure 3D). We found that neither wild type MEKK1 nor the PHD mutant significantly colocalize with lysosomes. To investigate whether the subcellular localization of MEKK1 or any of the MEKK1 mutants change upon treatment with MTIs, transfected HeLa cells were treated with vinblastine and subjected to immunofluorescence as before (Figure 3E). Within one hour of treatment both wild type MEKK1 and the kinase dead mutant changed localization, from diffuse/punctate in the periphery to aggregates in the peri-nuclear region. However, the appearance and location of the PHD mutant did not change after vinblastine treatment.

### The PHD mutant exhibits decreased ubiquitination

**Figure 4:**
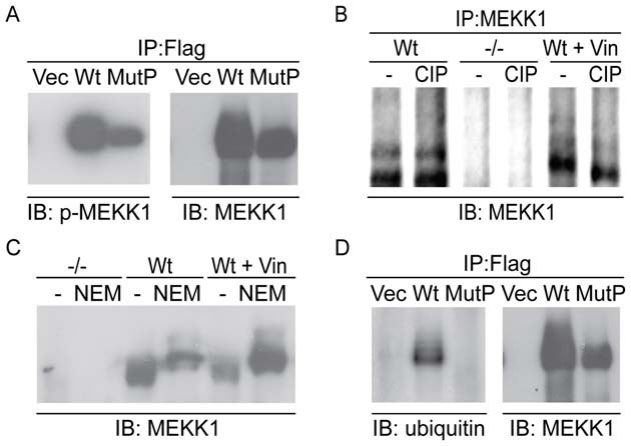
Identifying the post-translational modifications of the MEKK1 protein. *A*. The PHD mutant can be phosphorylated. Vector, wild type flag-MEKK1 and flag-PHD mutant (mutP) were transfected into 293T cells and immunoprecipitated with αflag M2 conjugated beads. Membranes were probed with total or phospho-MEKK1. *B*. CIP treatment does not affect basal MEKK1 modification, but does shift the molecular weight of vinblastine treated (phosphorylated) endogenous MEKK1. DT40 cells were treated with vinblastine for six hours and lysates were immunoprecipitated with αMEKK1. Half of each lysate was treated with calf alkaline phosphatase and all were incubated at 37°, run on SDS-PAGE gel and membranes were probed with αMEKK1. *C*. Inhibition of de-ubiquitinating enzymes via N-ethylmaleimide (NEM) stabilizes the higher molecular weight form of MEKK1. Wild type DT40 cells were lysed in modified RIPA. Lysates were treated with or without 20 mM NEM for 30 minutes at room temperature. Lysates were run on SDS-PAGE and probed with α MEKK1. *D*. MEKK1 is ubiquitinated whereas the PHD mutant is not. 293T cells were transiently transfected with vector, flag-MEKK1 or mutP. Cell lysates were immunoprecipitated with flag-conjugated beads, run on an SDS-PAGE gel and probed with αubiquitin or αMEKK1.

The difference in subcellular localization between wild type MEKK1 and the PHD mutant prompted us to investigate possible differences in post-translational modifications between the two proteins. Deak et al. have previously shown that overexpressed MEKK1 is auto-phosphorylated [22]. We hypothesized that the modified form of MEKK1 that we see in our assays (Figure 3B, MEKK1 upper bands) may be due to phosphorylation of multiple sites in MEKK1. To test this hypothesis, we set out to determine whether the loss of basal modification on the PHD mutant (lack of upper band in MutP lane, Figure 3B) correlated with the loss of MEKK1 phosphorylation. We found that both wild type MEKK1 and the PHD mutant were phosphorylated in the kinase domain when overexpressed (Figure 4A). Furthermore, treatment of DT40 cells with calf-alkaline phosphatase shifted the size of vinblastine treated (and therefore phosphorylated), but not untreated DT40 cells (Figure 4B), indicating that the observed basal modification on endogenous MEKK1 is not phosphorylation. However, the PHD also has intrinsic E3 ligase activity and destroying this domain results in defective auto-polyubiquitination [14]. Therefore, it is possible that the PHD is required for ubiquitination of MEKK1. Thus, we asked whether this modification can be due to ubiquitination. To determine if MEKK1 is ubiquitinated, we questioned whether the modification found on MEKK1 would be stabilized by a deubiquitinase inhibitor, such as N-ethylmaleimide (NEM). We found that the high molecular weight band of MEKK1 is enhanced by treatment of cell lysates with NEM when compared to untreated cells (Figure 4C). Finally, overexpression and immunoprecipitation of empty vector, MEKK1 or the PHD mutant followed by detection with ubiquitin antibody indicate that only wild type MEKK1 is strongly ubiquitinated (Figure 4D). These biochemical assays indicate that while the PHD mutant can be phosphorylated when overexpressed, modification by ubiquitin is abrogated in the PHD mutant.

### Endogenous level reconstitutions of MEKK1-deficient cells indicate that the PHD and the kinase domain of MEKK1 are required for JNK activation after cytoskeletal disruption

**Figure 5:**
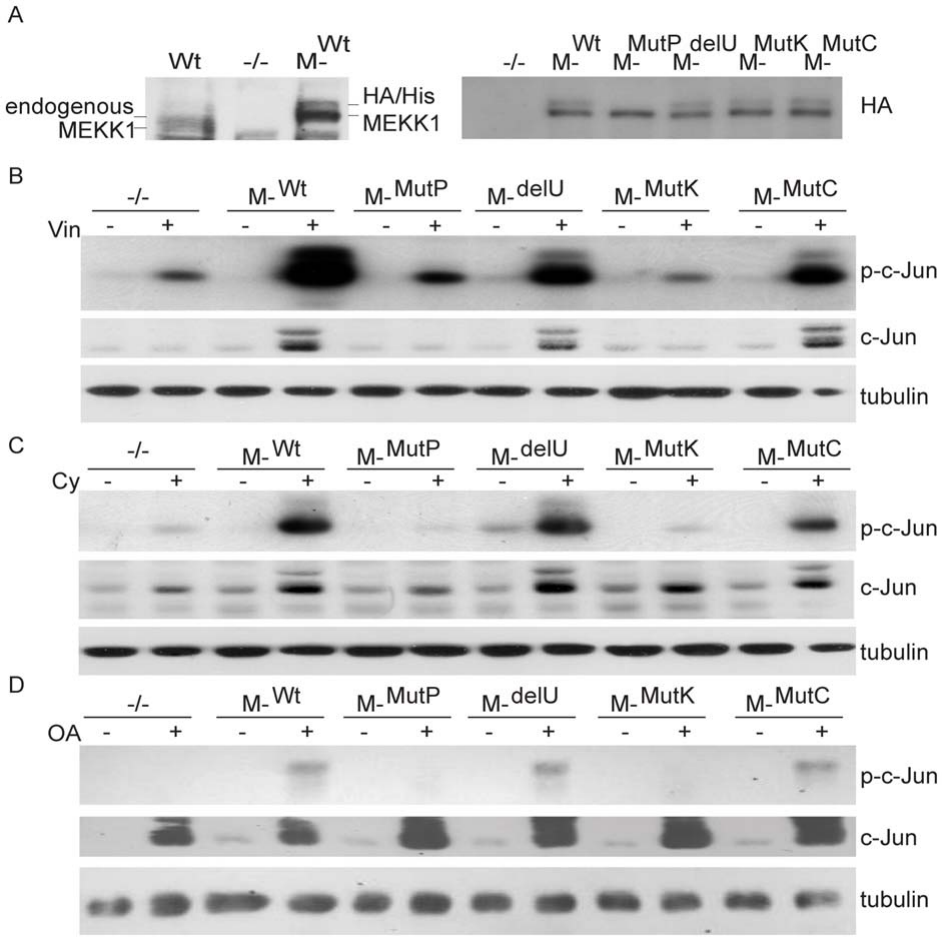
The PHD and kinase domains are essential for MEKK1-dependent JNK activation. *A*. Expression of mutations in relation to endogenous MEKK1. HA-His-tagged pBabeMEKK1 was introduced into *MEKK1^−/−^* (−/−) DT40 cells by retroviral infection (reconstituted cells are referred to here as M-^Wt^ and MEKK1^Wt^ in the text), selected with puromycin and protein levels were assessed by western blot probed with αMEKK1 (left panel). The size difference between His-HA-tagged and endogenous MEKK1 is indicated. Each mutation was introduced into *MEKK1* ^−/−^ cells and expression was compared (right panel). Reconstituted cell lines are referred to as M-^mutation^ within each figure. *B*. The PHD and kinase domain are required for c-Jun phosphorylation after vinblastine stimulation. Reconstituted cell lines were stimulated with 1 µM vinblastine (Vin) for four hours and lysed in modified RIPA. Phosphorylated c-Jun, total c-Jun and β-tubulin were assessed by western blot. *C*. There is a decreased amount of c-Jun phosphorylation in the *MEKK1* ^−/−^, PHD and kinase domain mutant cell lines after cytochalasin D treatment. Phosphorylation of c-Jun and total c-Jun levels of each reconstituted cell line was assessed by western blot after four hours of 2 µg/ml cytochalasin D (Cy) treatment. *D*. There is a decreased amount of c-Jun phosphorylation in the *MEKK1^−/−^*, PHD and kinase domain mutant cell lines after okadaic acid treatment. Phosphorylation of c-Jun and total c-Jun levels of each reconstituted cell line was assessed by western blot after four hours of 90 nM okadaic acid (OA) treatment.

To determine if any candidate domains were required for JNK activation in response to cytoskeletal disrupting drugs, each MEKK1 mutant was expressed in MEKK1-deficient DT40 cells. However, because the increased JNK and ERK activation caused by MEKK1 overexpression may interfere with the response to cytoskeletal disruption, we sought to first establish a low-level expression system using the pBabe retroviral vector, which would not significantly increase JNK or ERK activation in resting cells. After retroviral infection and selection with puromycin, the polyclonal populations of knockout cells reconstituted with MEKK1 were checked for expression levels in comparison to the DT40 cell line by western blot (Figure 5A, left panel). Murine MEKK1-deficient cells were not used due to inherent puromycin resistance that prevented the analysis of puromycin-selected polyclonal populations. While the level of expression was slightly higher in the reconstituted cell line, it was far less than traditional overexpression vectors. Furthermore, all reconstituted cells are shown to have relatively equivalent levels of MEKK1 mutant expression (Figure 5A, right panel). Additionally, reconstitution of the MEKK1-deficient cells did not show the increased basal MAPK activity seen when MEKK1 is overexpressed in the 293T system (Figure 5B, 5C, 5D).

Next, the MEKK1-deficient reconstituted cells were treated with cytoskeletal disrupting drugs, and the effects of these mutations on downstream signaling were determined. Cytoskeletal structure was disrupted with either 1 µM vinblastine, 2 µg/ml cytochalasin D or 90 nM okadaic acid for 4 hours and phosphorylation of c-Jun was analyzed by western blot. Robust phosphorylation of c-Jun was observed in the MEKK1^Wt^, MEKK1^mutC^ and MEKK1^delU^ reconstituted cell lines, but MEKK1^mutP^, MEKK1^mutK^ (MEKK1-deficient cells reconstituted with each mutation are referred to as MEKK1^mut^) and MEKK1-deficient cell lines demonstrate abrogated c-Jun phosphorylation in response to cytoskeletal disruption. Total levels of c-Jun were also analyzed and levels were enhanced after cytoskeletal disruption (Figure 5B, C and D). This increase appears to be partially dependent on MEKK1 activation, but the extent of MEKK1 dependence seems to vary depending on the stimuli.

**Figure 6:**
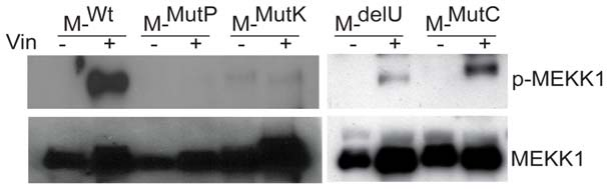
MEKK1^mutP^ is not phosphorylated in the activation loop after vinblastine treatment. *MEKK1^−/−^*, MEKK1^Wt^, MEKK1^mutP^ or MEKK1^mutK^ (left panel) and MEKK1^delU^, MEKK1^mutC^ (right panel) cell lines were treated with 1 µM vinblastine for 2 hours. Cell lysates were immunoprecipitated with αHA-agarose, run on SDS-PAGE gel and probed with αphospho-MEKK1 followed by detection of total MEKK1 with αMEKK1 C-22.

After determining that the MEKK1^mutP^ cell line exhibits decreased amounts of c-Jun phosphorylation after cytoskeletal disruption, the cause of this defect was investigated. We had already observed that MEKK1^mutP^ can activate downstream signaling when overexpressed (Figure 3B), so we hypothesized that the defect in activation of the MEKK1^mutP^ cell line (where MEKK1 is expressed at near endogenous levels and therefore cannot auto-activate downstream signaling) lies in receiving upstream signals. To test this we investigated whether MEKK1 can become phosphorylated after treatment with vinblastine within the active site of its kinase domain, which also corresponds to activation of this protein as a kinase [15], [23]. We treated knockout cells reconstituted with MEKK1 mutant cell lines with vinblastine and subjected the lysates to immunoprecipitation, western blot, and detection with a phosphorylation-specific antibody indicative of MEKK1 activation [23]. We determined that within 2 hours of vinblastine treatment MEKK1^Wt^, MEKK1^delU^ and MEKK1^mutC^ are active as indicated by auto-phosphorylation, whereas MEKK1^mutP^ and MEKK1^mutK^ are not (Figure 6), indicating that mutation of the PHD results in an inability to be activated under physiologically relevant conditions.

**Figure 7:**
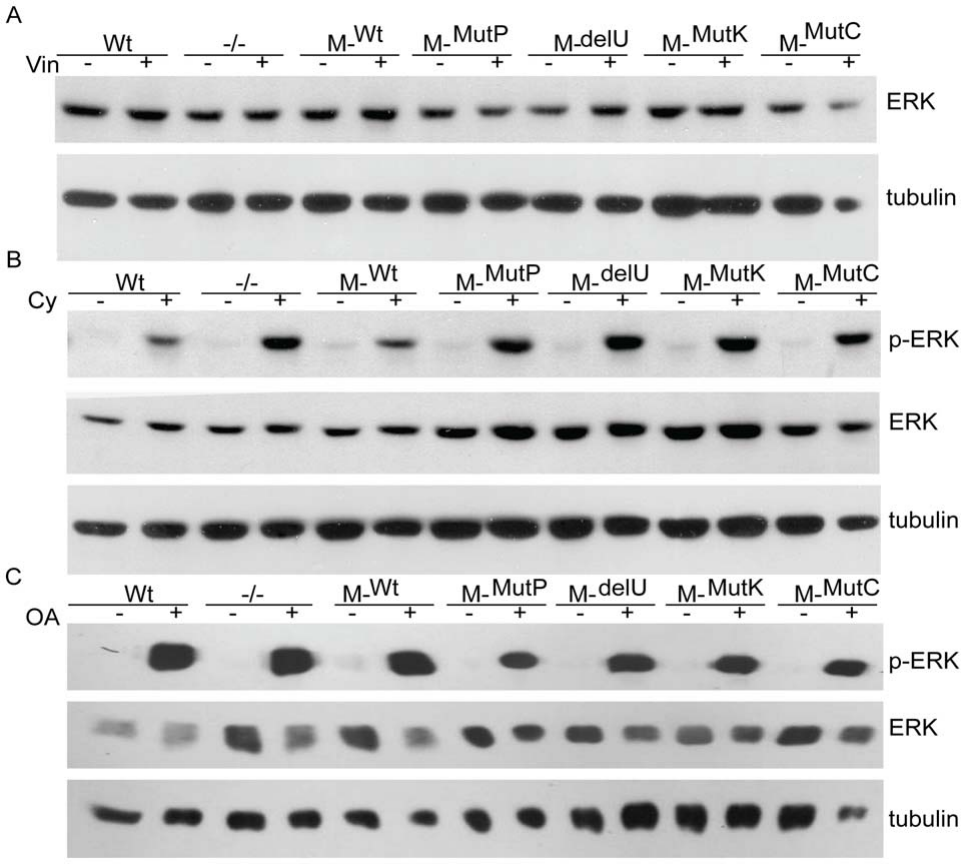
The PHD and kinase domains of MEKK1 are not essential for maximal ERK activation. *A*. ERK is not activated or degraded after vinblastine treatment. Wild type, knockout and MEKK1-reconstituted *MEKK1^−/−^* cells were treated with 1 µM vinblastine for four hours and total ERK or β-tubulin were assessed by western blot. *B*. Activation of ERK by cytochalasin treatment is not MEKK1-dependent. Cell lines were treated with 2 µg/ml cytochalasin D for two hours and phosphorylated and total ERK were evaluated by western blot. *C*. Activation of ERK by okadaic acid treatment is not MEKK1-dependent. Phosphorylation of ERK and total ERK were determined by western blot after treatment with 90 nM okadaic acid for four hours. β-tubulin was used as a loading control.

Because MEKK1 can degrade ERK via the activity of the PHD under conditions of osmotic stress, and ERK activation is generally linked to survival signals, ERK levels and its activation were also analyzed after cytoskeletal disruption [11], [13]. As with the parental DT40 and MEKK1-deficient cells, vinblastine treatment did not change the total level of ERK (Figure 7A), and ERK phosphorylation was undetectable. While ERK was activated from 30 minutes to six hours after cytochalasin D and okadaic acid treatment, no differences were seen between the wild type, knockout and reconstituted lines (Figure 7, B and C).

### The PHD domain and the kinase domain are required for apoptosis after cytoskeletal disruption

**Figure 8:**
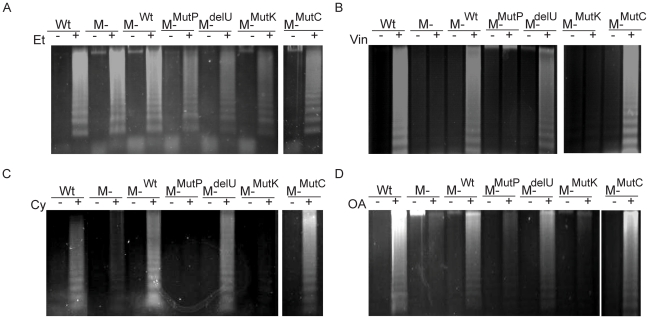
The PHD and kinase domains are required for vinblastine induced apoptosis. *A*. *MEKK1^−/−^* DT40 cells reconstituted with MEKK1 or mutations thereof all initiate apoptosis in response to etoposide. Each reconstituted cell line was treated with 25 µM etoposide (Et) for four hours and subjected to DNA laddering assay. *B*. *MEKK1^−/−^* cell lines reconstituted with MEKK1 containing the PHD or kinase mutations do not result in DNA fragmentation after vinblastine treatment. Each reconstituted cell line was treated with 1 µM vinblastine (Vin) for six hours and subjected to a DNA laddering assay. *C*. There is a defective the DNA laddering response in the PHD or kinase domain mutant cell lines after cytochalasin D treatment. Reconstituted cell lines were treated with 2 µg/ml cytochalasin D (Cy) for six hours and subjected to DNA laddering assays. *D*. There is a defective the DNA laddering response in the PHD or kinase domain mutant cell lines after okadaic acid treatment. Reconstituted cell lines were treated with 90 nM okadaic acid (OA) for six hours and subjected to DNA laddering assays.

**Figure 9:**
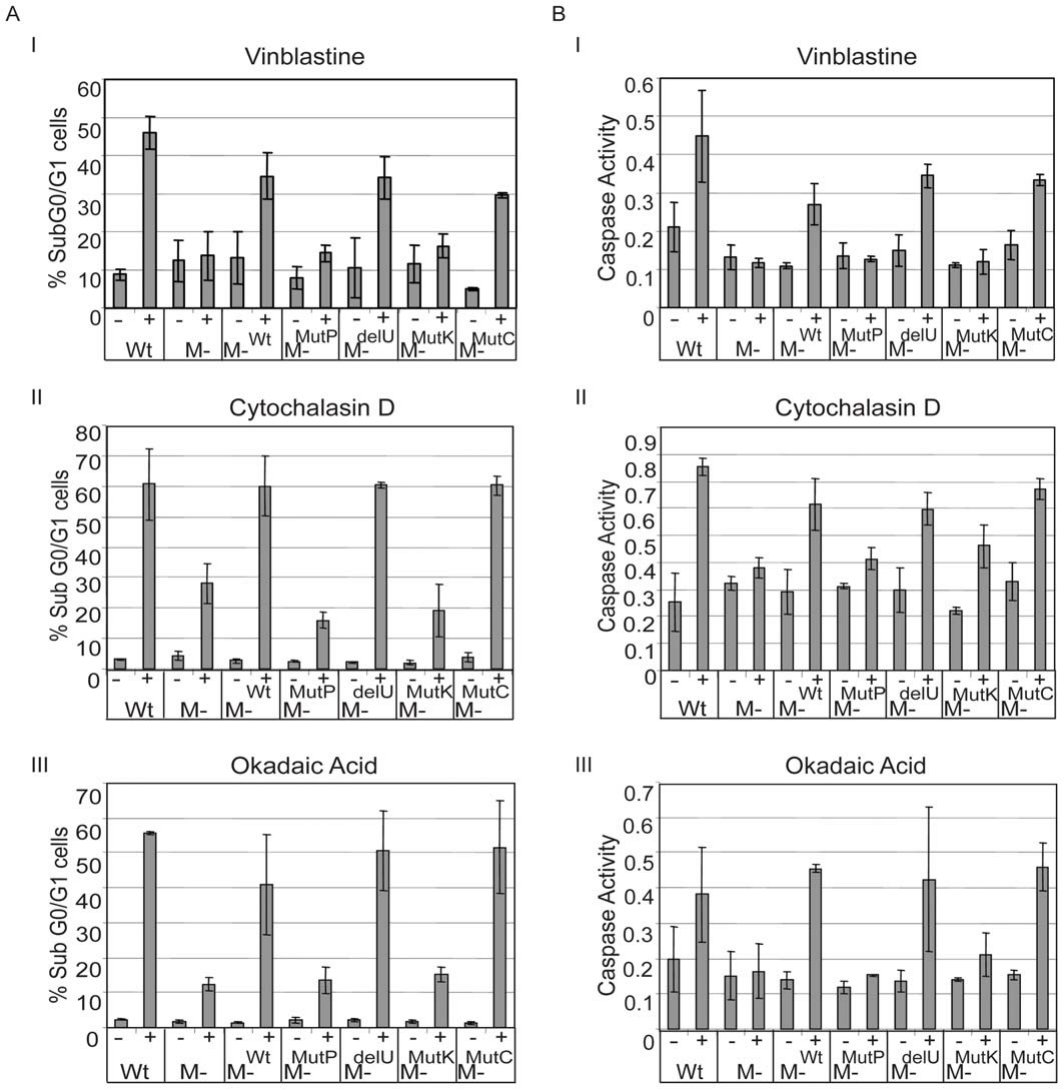
Quantification of apoptosis by propidium iodide incorporation and caspase 3 activity. *A*. In the MEKK1^mutP^ and MEKK1^mutK^ cell lines the percentage of apoptotic cells does not significantly increase after cytoskeletal disruption. To quantify the levels of DNA fragmentation, propidium iodide staining was analyzed by flow cytometry. Cell lines were treated with 1 µM vinblastine (panel I), 2 µg/ml cytochalasin D (panel II) or 90 nM okadaic acid (panel III) for twelve hours and the percentage of apoptotic cells were determined by measuring fluorescence lower than the diploid population using CellQuest software. *B*. Caspase 3 is not activated in the MEKK1^mutP^ or MEKK1^mutK^ cell lines after cytoskeletal disruption. Caspase 3 activity was determined after four hours of treatment with 1 µM vinblastine (I), 2 µg/ml cytochalasin D (II) or 90 nM okadaic acid (III) treatment. Cell lysates were used to determine the catalytic activity of caspase 3 by using the colorimetric substrate Ac-DEVD-pNA and measured at 405 nM.

To identify the domains of MEKK1 required for apoptosis induced by cytoskeletal disruption, DT40 reconstituted cell lines were stimulated with vinblastine, cytochalasin D or okadaic acid, and apoptosis was analyzed via DNA laddering (Figure 8B, C and D). While wild type DT40 cells, MEKK1^Wt^, MEKK1^delU^ and MEKK1^mutC^ reconstituted cells exhibited DNA laddering after cytoskeletal disruption, the MEKK1^mutP^ and MEKK1^mutk^ reconstituted cells were resistant to apoptosis. This defect in apoptosis was not the result of general resistance to apoptosis, as each cell line could still induce apoptosis in response to etoposide, a MEKK1-independent stimulus (Figure 8A). The response to cytoskeleton disruption was then quantified by PI exclusion assay, which is consistent with the DNA laddering results (Figure 9A). Our results show that MEKK1, MEKK1^delU^ and MEKK1^mutC^ induced apoptosis at levels similar to wild type cells, but there was no significant increase in the percentage of apoptotic cells in the knockout, MEKK1^mutK^ or MEKK1^mutP^ cell lines with vinblastine, cytochalasin D or okadaic acid (Figure 9A panels Ι, ΙΙ, ΙΙΙ, respectively). Vinblastine-induced apoptosis has been shown to activate the caspase pathway and Figure 9B shows that caspase 3 activity is also significantly reduced in the MEKK1^mutP^ and MEKK1^mutK^ as compared to the MEKK1^Wt^, MEKK1^delU^ and MEKK1^mutC^ reconstituted cells after treatment with vinblastine, cytochalasin D or okadaic acid (Figure 9B panels Ι, ΙΙ, ΙΙΙ, respectively).

## Discussion

MTIs such as vinblastine are important chemotherapy drugs, but the mechanism by which these drugs initiate apoptosis is not clear. We have previously shown that MEKK1 is required for apoptosis in DT40 B cells in response to MTIs [2]. However, the requirement of MEKK1-dependent apoptosis upon treatment with other toxic agents had not been thoroughly investigated. Here we have shown that MEKK1 activation is involved in apoptosis induced by general cytoskeleton disruption and that a functional PHD and kinase domain are required to induce apoptosis.

The requirement of MEKK1 for JNK activation in response to MTIs has been consistent in published data. However, in different studies the outcome of JNK activation after treatment with microtubule inhibitors has been attributed to two opposing effects, either enhanced apoptosis or increased cell survival Our previous study with DT40 cells indicates that MEKK1 is required for JNK activation and apoptosis after vinblastine stimulation. However, it has been published that treatment of *Mekk1^−/−^* ES cells with the MTIs nocodazole or taxol result in defective JNK activation but somewhat increased sensitivity to apoptosis [6], [19], [24]. Whether this discrepancy was due to a cell-specific effect or a species-specific effect was unclear. Here we report that vinblastine-stimulated apoptosis is MEKK1-dependent in both chicken DT40 and murine B cell lines, indicating that the discrepancy is not specific to chicken cells.

In order to further investigate how MEKK1 functions within this apoptotic pathway, we created mutations in characterized domains within MEKK1 and reconstituted MEKK1-deficient DT40 cells at near endogenous levels, which would not independently activate MAPK pathways and confound our results. By expressing each mutation at levels that do not increase JNK or ERK activation in resting cells, we were able to clarify which domains of MEKK1 are actually involved in carrying out apoptosis after cytoskeletal disruption. Among the four mutations in our study, the caspase cleavage and UIM deletion mutants were able to compensate for MEKK1 in our readout assays, indicating that these domains are unnecessary for MEKK1 function after cytoskeletal disruption. The caspase recognition site has been postulated to be required for the release of the kinase domain from a particulate fraction to a soluble cytoplasmic fraction, which was shown to be necessary for the pro-apoptotic function of MEKK1 in an overexpression system [21]. Previously, in a 293T overexpression system, Widmann et al. showed that mutation of the caspase 3 recognition site rendered murine MEKK1 less effective in inducting apoptosis caused by overexpression of MEKK1 [17]. However, in our system the orthologous mutation in the rat MEKK1 caspase site is inconsequential for vinblastine-induced JNK activation and apoptosis (Figure 5 and 9). As seen in Figures 8 and 9, reconstituted DT40 cells expressing the MEKK1^mutc^ at near endogenous levels undergo apoptosis after treatment with cytoskeletal disrupting drugs. In addition, downstream signaling as detected by c-Jun phosphorylation is unaffected (Figure 5B, C and D). There are a number of possible explanations for this apparent discrepancy. First, in our system with treatment of cytoskeletal disrupting drugs the cytoskeleton structure is already disrupted and MEKK1 moves to a region near the nucleus (Figure 3E). This movement is also detected by antibodies to the N-terminal flag tag on flagMEKK1 (data not shown); therefore MEKK1 cleavage and kinase domain release from the particulate fraction are not necessary for MEKK1 movement after cytoskeletal disruption. It is also possible that in the 293T overexpression system used by Widmann et al., the caspase cleavage mutant is highly overexpressed and therefore could function in a manner different than endogenous-level MEKK1 [17]. Additionally, our studies do not rule out the possibility of alternative cleavage sites in the chicken B cells that could compensate for mutation of the characterized caspase cleavage site.

Deletion of the UIM domain also does not affect MEKK1-dependent JNK activation, apoptosis or modification in our B cell reconstitution system. UIM regions contained in other proteins have been shown to directly interact with ubiquitin and can enhance the ubiquitination of these UIM-containing proteins [16], [25]. For example, auto-ubiquitination of TRAC-1 requires both its RING and UIM domains, as point mutations in either domain reduce the amount of ubiquitinated TRAC-1 [25]. MEKK1 is also autoubiquitinated when overexpressed in conjunction with ubiquitin. Ubiquitination of endogenous MEKK1 occurs after CD40 stimulation composed of K63-linked (activation related) ubiquitin chains [15], [23], [26]. However, the function of MEKK1 ubiqutination has not been further investigated. We hypothesized that the UIM region was required for either monoubiqutination or polyubiquitination of MEKK1, and through these actions, it can influence MEKK1 activation. The previous study regarding the function of the UIM within MEKK1 used a 35 kDa isolated region of MEKK1 containing the UIM linked to yellow fluorescent protein (YFP) to examine its ability to interact with ubiquitin [27]. It was shown that while other UIM regions such as Hrs or epsin were ubiquitinated, the MEKK1 UIM region was unable to bind ubiquitin. The lack of interaction of MEKK1 UIM and ubiquitin in this study could have been due to the fact that the overall tertiary structure of MEKK1 is necessary for the function of the MEKK1 UIM, so in our experiments, we deleted the entire 16 amino acid region containing the UIM while retaining all other domains of full length MEKK1. However, the UIM deletion mutant of MEKK1 was able to function similarly to wild type MEKK1, indicating this domain is unnecessary for MEKK1-dependent cell death by cytoskeletal disruption. We determined that the UIM mutant can be auto-ubiquitinated in an overexpression system to the same extent as wild type MEKK1 (data not shown). Our data, combined with the YFP-UIM data, suggests that while the consensus sequence for a UIM region is present in MEKK1, this domain is not absolutely required for kinase activation or autoubiquitination of MEKK1.

In this study, JNK activation correlates with apoptosis after treatment with cytoskeletal disrupting drugs. In our MEKK1-deficient cells, JNK activation is abrogated, and reconstitution of these cells with MEKK1 restores JNK activation. Therefore, JNK activity is used as an indicator of MEKK1 activity. In this paper and our previous study, MEKK1-dependent JNK activation correlates strongly with apoptosis, and previous reports have shown that absence or inhibition of JNK resulted in decreased levels of apoptosis with vinblastine treatment [28], [29]. We also show that the JNK substrate c-Jun upregulation after vinblastine treatment is partially dependent upon MEKK1, and upregulation of c-Jun has been shown to contribute to vinblastine-induced apoptosis [30]. The duration of JNK activation may also be important for inducing apoptosis. Here, vinblastine, cytochalasin and okadaic acid treatment all result in steady, MEKK1-dependent JNK activation until cell death, while activation of other potential MEKK1 targets such as ERK, p38 or NF-κB are unaffected by the loss of MEKK1 [2]. In other systems, prolonged JNK activation has been linked to enhancing apoptosis [1]. For example, short term activation of JNK after TNFα stimulation results in proliferation and survival, but TNFα treatment with conditions resulting in prolonged JNK activation promotes cell death [31], [32]. Given that the kinase activity of MEKK1 is required for cell death, JNK is robustly activated by MEKK1, and that prolonged JNK activation has been tied to cell death, it is strongly suggested that JNK activation plays a significant role in MEKK1-dependent apoptosis in response to cytoskeletal disrupting drugs.

Mutations of both the PHD and kinase domains reduce JNK activation and abrogate MEKK1-dependent apoptosis after cytoskeletal disruption. While disrupting the PHD domain at physiologically relevant levels clearly results in defective JNK activation and apoptosis (Figures 5 and Figure 8–9, respectively), we and others have shown that overexpressing MEKK1 with a mutated PHD at high levels results in activation of and possibly enhanced downstream signaling [14]. Therefore, it is possible that the PHD is required for specific upstream interactions that are unnecessary in overexpression systems. There is also evidence that mutation of the PHD results in the inability to interact with MEKK1 activating proteins such as RhoA or TRAF2, which interact with MEKK1 after activin or CD40 ligand stimulation [12], [15]. However in our system these particular interactions do not appear to be required for the MEKK1-dependent apoptosis. Overexpression of a dominant negative RhoA in 293T cells did not inhibit vinblastine-induced JNK activation. Furthermore, TRAF2-deficient mouse embryonic fibroblasts (MEFs) exhibit normal JNK activation after vinblastine treatment, indicating they are not upstream of MEKK1 in the vinblastine-activated pathway (data not shown).

Other functions for the PHD within MEKK1 have been proposed. Lu et al. and Xia et al. have shown that the PHD is required for MEKK1 to ubiquitinate and degrade ERK and c-Jun under conditions of osmotic stress [11], [13]. Although we can repeat this data with our system when under hyperosmotic conditions (data not shown), after vinblastine treatment ERK and c-Jun are not degraded (Figure 7 and Figure 5 B, C and D). While we do not detect ubiquitination and degradation of MEKK1 substrates, we do see that MEKK1^mutP^ lacks the modification normally seen with MEKK1 (Figure 3B and Figure 5A). We have performed multiple biochemical assays which indicate this modification is ubiquitin (Figure 4C and D). Since ubiquitination has been reported to affect protein interactions as well as protein sorting and trafficking, we hypothesized that decreased ubiqutination of the PHD mutant results in the mislocalization of MEKK1. The localization of MEKK1 has been well characterized by immunofluorescence [21]. Our data confirms that MEKK1 is expressed diffusely through the cytosol and in small punctuate structures (Figure 3C). However, the PHD mutant presents a strikingly different localization than wild type MEKK1. We further illustrate that this localization is not due to enhanced lysosomal degradation by immunofluorescence with a lysosome marker, LAMP1 (Figure 3D). The LAMP1 protein associates with late endosomes/lysosomes and when detected in conjunction with MEKK1 or the PHD mutant, we did not observe a difference in co-localization. Interestingly, both wild type MEKK1 and kinase dead mutant shift localization to near the nucleus after vinblastine treatment, (Figure 3E), whereas the PHD mutant does not change its cellular localization pattern after cytoskeletal disruption. Thus, this alteration in cellular localization appears to be independent of MEKK1 activity, supporting the possibility that mutation of the PHD results in aberrant localization due not to impaired activation after vinblastine treatment, but from a change in initial protein interactions. Since ubiquitination can alter protein localization, lack of ubiquitination may explain mislocalization caused by mutation of the PHD and the importance of the PHD for interaction with many proteins, but this requires further investigation.

Finally, we have shown that MEKK1-dependent apoptosis occurs not only by MTIs, but also in apoptosis induced by actin disruption and protein phosphatase inhibition (Figure 2). Actin disruption can activate ERK and JNK and induce apoptosis in lymphocytes, airway epithelial cells and various cancer cell lines [33], [34], [35]. Okadaic acid and another protein phosphatase inhibitor, cantharidin, also induce apoptosis in lymphocytes and cause extensive activation of the JNK, ERK and p38 MAPK pathways [36], [37]. While cytochalasins, protein phosphatase inhibitors and microtubule inhibitors have diverse effects upon cells, maximal JNK activation and apoptosis are only triggered in the presence of MEKK1. In this study we have shown that effective treatment with three different classes of drugs requires MEKK1. Therefore, screening more drug panels for prolonged MEKK1/JNK activation may result in the identification of novel chemotherapeutics or combinations of existing drugs.

It is critical to understand the MEKK1-dependent apoptotic pathway, as vinblastine and other vinca alkaloid family members play a key role in chemotherapy for lymphomas, leukemia, breast, testicular and some non-small cell lung cancers. To date, most recommended chemotherapy regimens use combinations of drugs, and understanding how MEKK1 activity is affected by these drug combinations could enhance the efficacy of therapy. For example, drug combinations that result in higher MEKK1 activity may be desirable for treatment of leukemia and lymphoma, which induce rapid cell death via MEKK1. Further study of the MEKK1-dependent pathway of apoptosis will be useful in targeting new molecules for cancer therapy, and for understanding resistance to cytoskeletal disruption-induced apoptosis.

## Materials and Methods

### Ethics Statement

The human cell lines used in this study are well established and were handled under the biosafety level 2 standards established by UCLA. The murine B cell lines were created from MEKK1 wild type and knockout murine bone marrow, shipped to us from the laboratory of Bing Su at Yale University, using the standard p210 BCR-ABL retroviral transformation protocol complicit with biosafety level 2 standards. As this study did not require direct work with mice or primary human tissues, no additional approval was necessary.

### Plasmids

PBabe ratMEKK1 containing a hemagglutinin (HA) and a His tag was generated by digesting pskMEKK1+EcoR1 plasmid with EcoR1 and subcloning into the Maloney Murine Leukemia Virus-based retroviral vector pBabepuro vector. PApuro ratMEKK1 was generated by excising MEKK1 from the pCEP4HAMEKK1 vector and this insert was subcloned into the chicken actin promoter expression vector pApuro between Spe1 and Sal1 restriction enzymes sites in the polylinker. MEKK1 mutations were created by PCR mutagenesis. N-terminal flag-tagged MEKK1 was generated by subcloning into the CMV-flag construct provided generously by the lab of Steve Smale.

### Reagents

Vinblastine sulfate was acquired from MP Biomedicals (Solon, OH). Cytochalasin D and okadaic acid were obtained from Sigma-Aldrich (Saint Louis, MO). FuGENE 6 was purchased from Roche Molecular Biochemicals (Indianapolis, IN). Lipofectamine 2000 was purchased from Invitrogen. Anti-HA Y-11 and Anti-MEKK1 were purchased from Santa Cruz Biotechnologies (Santa Cruz, CA). Anti-phosphorylated c-Jun, anti-c-Jun, anti-phosphorylated ERK1/2 and anti-ERK1/2 were purchased from Cell Signaling Technologies (Beverly, MA). Anti-HA was purchased from Covance (Cumberland, VA). Anti-β tubulin, αflag (M2), αflag-conjugated agarose beads and EZ view HA-conjugated agarose beads were obtained from Sigma-Aldrich (Saint Louis, MO). Protein G sepharose fast flow 4 was purchased from GE Healthcare Biosciences (Piscataway, NJ). The phospho-MEKK1 antibody was generously donated by Dr. Ewen Gallagher. This antibody has been shown to recognize active MEKK1 [15], [23]. The CaspACE 3 assay was obtained from Promega (Madison, WI). FITC secondary antibodies were purchased from Caltag (Burlingame, CA) and Texas red-phalloidin from Molecular Probes. The goat anti-rabbit IgG horseradish peroxidase was obtained from (Santa Cruz Biotechnology, Inc).

### Cell culture and transfections

HEK 293T cells were maintained with Dulbecco's modified Eagle's medium (DMEM) supplemented with 10% fetal bovine serum (FBS), 100 IU/ml penicillin, and 100 µg/ml streptomycin. Transient transfections were performed using FuGENE 6 according to the manufacturer instructions. DT40 and murine B cell lines were maintained in DT40 medium (RPMI 1640 medium containing 10% FBS, 1% chicken serum, 50 µM 2-mercaptoethanol, 50 U of penicillin per ml, and 50 µg of streptomycin per ml) at a density of 0.5×10^6^ to 1.5×10^6^ cells/ml. HeLa cells were maintained in DMEM with 5% FBS and 100 IU/ml penicillin and 100 µg/ml streptomycin.

### Creation of murine B cell line

A murine stem cell virus vector containing the BCR-Abelson fusion gene and GFP was transfected with helper virus plasmid into HEK 293T cells, and supernatant was harvested after 36 hours. The filtered supernatant was used to infect bone marrow cells from either wild type or MEKK1-deficient mice from the lab of Bing Su. After approximately three weeks B cell populations arose and clones were selected, as previously described [38].

### *MEKK1^−/−^* reconstitution

Viral transduction was used to reconstitute DT40 MEKK1-deficient cells; briefly, HEK 293T cells were transfected with a viral helper construct plus pBABEpuro containing the indicated MEKK1 construct, using standard calcium phosphate methods. MEKK1-deficient cells were then infected with the filtered 293T cell supernatants followed by selection with 0.4 µg/ml of puromycin.

### Western blotting and immunoprecipitation

MEKK1 and MAPK western blotting was previously described [2]. Briefly, 5×10^6^ cells were lysed in modified RIPA or lysed directly in 1× SDS loading buffer. Samples were sonicated, and 25 µg or 75 µg of protein were boiled in SDS loading dye and run on a 10% or 7% polyacrylamide electrophoresis separating gel respectively for MAPK or MEKK1. The protein was then transferred onto nitrocellulose membrane and then probed with the indicated antibody. For immunoprecipitation of DT40 cell lines, 50×10^6^ cells were resuspended in lysis buffer (20 mM Tris-Hcl pH 7.5, 150 mM NaCl, 1% Triton-X 100 and 2 mM EDTA supplemented with protease inhibitors, 5 mM NaVO_4_ and 1 mM NaF). Cells were sonicated and lysates were cleared by centrifugation. 20 µl of HA-agarose beads were added to the lysates and incubated overnight at 4°C. Samples were then washed four times with lysis buffer, boiled in SDS loading buffer, run on SDS-PAGE gel and protein was detected by anti-phosphorylated or total MEKK1 antibody. For immunoprecipitation from transiently transfected 293T cells, 20 µl of flag-conjugated beads were added to cell lysates instead of HA-agarose beads.

### Analysis of Apoptosis

DNA laddering assays were performed as previously described [2]. Briefly, 3.75×10^6^ wild type DT40 or MEKK1-deficient cells were treated with 1 µM vinblastine sulfate, 2 µg/ml cytochalasin D or 90 nM okadaic acid and then processed as previously described. Propidium iodide staining protocol was followed as previously described [39]. In brief, cells were stimulated with 1 µM vinblastine sulfate, 2 µg/ml cytochalasin D or 90 nM okadaic acid for the indicated time points, washed once with PBS and resuspended in Nicoletti buffer (0.1% sodium citrate and 0.1% Triton-X 100) and fluorescence intensity was analyzed via flow cytometry. In the caspase 3 activity assay, 2×10^7^ cells treated for the indicated times with 1 µM vinblastine sulfate, 2 µg/ml cytochalasin D or 90 nM okadaic acid, were lysed in 50 µl of ice-cold cell lysis buffer containing 50 mM HEPES (pH 7.4), 100 mM NaCl, 0.1% 3-[(3-cholamidopropyl)-dimethylammonio]-1-propanesulfonate (CHAPS), 1 mM dithiothreitol, and 0.1 mM EDTA, flash frozen in an ethanol-dry ice bath, and stored at −80°C before use. Caspase 3 activity assay experiments were performed as described in the Calbiochem protocol for colorimetric caspase 3 substrate I (Ac-DEVD-pNA). *A*_405_ readings were taken on a plate reader at four hour intervals.

### Immunofluorescence

To assess MEKK1 localization, HeLa cells were used due to superior surface adhesion and high transfection efficiency. HeLa cells were grown on coverslips and transfected using FuGENE6 according to the standard protocol. Twenty four hours post transfection cells were fixed with cold methanol for three minutes or 4% paraformaldehyde for 12 minutes, washed 3 times with PBS and then permeabilized with 0.1% Triton-X 100 in PBS for 3 minutes. Primary antibodies were used at a 1∶300 dilution in PBS with 3% BSA overnight at 4° and washed 3 times with PBS for 5 minutes. Secondary antibodies were conjugated to either FITC or TRITC and used at a dilution of 1∶1000 in PBS with 3% BSA.

### CIP treatment

DT40 cells were treated with 1 µM vinblastine for six hours and cells were lysed in modified RIPA. MEKK1 was immunoprecipitated with 4 µg αMEKK1C-22 and 40 µl protein G sepharose beads. Beads were resuspended in NEB buffer 3 with or without calf alkaline phosphatase and incubated for one hour at 37°C. SDS loading buffer was added and reactions run on SDS-PAGE. Membranes were probed with αMEKK1.
